# The Catalytic Domain Mediates Homomultimerization of MT1-MMP and the Prodomain Interferes with MT1-MMP Oligomeric Complex Assembly

**DOI:** 10.3390/biom12081145

**Published:** 2022-08-19

**Authors:** Marton Fogarasi, Simona Dima

**Affiliations:** 1 Cancer Research UK Cambridge Institute, Li Ka Shing Centre, Cambridge CB2 0RE, UK; 2 Center of Excellence in Translational Medicine, Fundeni Clinical Institute, 022328 Bucharest, Romania

**Keywords:** protein engineering, MT1-MMP, catalytic domain, oligomerization

## Abstract

Homomultimerization of MT1-MMP (membrane type 1 matrix metalloproteinase) through the hemopexin, transmembrane, and cytoplasmic domains plays a very important role in the activation of proMMP-2 and the degradation of pericellular collagen. MT1-MMP is overexpressed in many types of cancers, and it is considered to be a key enzyme in facilitating cancer cell migration. Since the oligomerization of MT1-MMP is important for its proteolytic activity in promoting cancer invasion, we have further investigated the multimerization by using heterologously expressed MT1-MMP ectodomains in insect cells to gain additional mechanistic insight into this process. We show that the whole ectodomain of MT1-MMP can form dimers and higher-order oligomeric complexes. The enzyme is secreted in its active form and the multimeric complex assembly is mediated by the catalytic domain. Blocking the prodomain removal determines the enzyme to adopt the monomeric structure, suggesting that the prodomain prevents the MT1-MMP oligomerization process. The binding affinity of MT1-MMP to type I collagen is dependent on the oligomeric state. Thus, the monomers have the weakest affinity, while the binding strength increases proportionally with the complexity of the multimers. Collectively, our experimental results indicate that the catalytic domain of MT1-MMP is necessary and sufficient to mediate the formation of multimeric structures.

## 1. Introduction

Membrane type 1 matrix metalloproteinase (MT1-MMP; MMP-14) is a zinc-dependent type I transmembrane endopeptidase with very important roles both in physiological (epithelial morphogenesis, skeletal development, and wound healing) and pathological (angiogenesis, arthritis, tumor growth, and metastasis, obesity, inflammation, and atherosclerosis) events. Initially, it has been identified by its ability to induce tumor cell invasion through proteolytic processing of proMMP-2 to its active MMP-2 form on the cell surface [1]. MT1-MMP is expressed on the surface of many types of cells: mesenchymal stem cells, fibroblasts, osteoblasts, osteoclasts, chondrocytes, epithelial cells, endothelial cells, adipocytes, myeloid cells, neuronal cells, T-cells, and B-cells [2]. It plays an important role in the remodeling of the extracellular matrix (ECM) by degrading a large number of extracellular molecules, such as collagen type I, II, III, fibronectin, laminin, vitronectin, fibrin, and aggrecan [3]. It sheds cell surface syndecan and CD44 receptors [4], and it is implicated in the activation of other matrix metalloproteinases including proMMP-2, proMMP-8, and proMMP-13 [5]. Pathologically, upregulated MT1-MMP is correlated with poor clinical outcomes, and it is involved in the tumor initiation, invasion, and metastasis of many types of cancers such as melanoma, pancreatic cancer, advanced neuroblastoma, small cell, and non-small cell lung cancer, mesothelioma, tongue squamous cell carcinoma, head and neck carcinoma, bladder cancer, breast cancer, colorectal cancer, and ovarian cancer [6]. High levels of the shedded soluble form of MT1-MMP were proposed as a biomarker that predicts poor survival of gastric cancer patients [7].

Structurally, MT1-MMP has a modular structure consisting of a prodomain, a catalytic domain bearing a Zn^2+^-binding sequence, a hinge/linker region that bridges the catalytic domain to the hemopexin domain, a stalk region, a transmembrane domain, and a short cytoplasmic tail. As this metalloproteinase is produced in the form of a zymogen, it requires the removal of the prodomain to be activated and perform its biological functions. At the junction of the prodomain and the catalytic domain, there are two sets of basic amino acids motifs: R^89^RPR^92^↓C^93^ and R^108^RKR^111^↓Y^112^ [8], which are recognized by furin-like proprotein convertases and can cleave off the prodomain during the secretion to the cell surface, thus resulting the catalytically active enzyme.

MT1-MMP biological functions are regulated through many cellular mechanisms, namely: gene expression, lysosomal proteolysis [9], homodimerization [10], ectodomain shedding [11], activation by proprotein convertases [8], cell surface proteolytic processing [12,13], inhibition by endogenous inhibitors [14,15], clathrin- and caveolae-dependent endocytosis [16,17], recycling to the cell surface [18], and palmitoylation [19]. Among these regulatory mechanisms, oligomerization represents an important and interesting pathway to investigate. Thus, it has been shown that MT1-MMP can oligomerize via several domains and each type of multimerization provides an important function. Reports demonstrated that the multimeric structures of MT1-MMP can be built up through the hemopexin domain [10] and transmembrane domain [20], or that there is a cooperation between hemopexin, cytoplasmic, and transmembrane domains [21]. Another report showed that the MT1-MMP can dimerize by forming a disulfide bridge between the cysteine residues located in the cytoplasmic tail [22].

Oligomerization of MT1-MMP has a significant impact on its biological functions. Homodimerization of MT1-MMP has a crucial role in cell surface collagenolysis at the leading edge of the migrating cell, thus generating a path for cell invasion [23,24]. In addition, MT1-MMP dimerization is necessary for the conversion of proMMP-2 into active MMP-2. Thus, activated MMP-2 facilitates the degradation of type IV collagen [25] in the basement membrane, as MT1-MMP cannot cleave this type of collagen [3].

In this report, we further investigate the mechanism of multimeric assembly of the MT1-MMP by using the heterologously expressed ectodomains in eukaryotic cells. We find that the catalytic domain of MT1-MMP is involved in the formation of oligomeric structures of the enzyme. Furthermore, our results show that a point mutation in the furin recognition motif inhibits the removal of the prodomain and, as a consequence, the MT1-MMP ectodomain is found in a monomeric state, suggesting that the presence of the prodomain interferes with the oligomerization process. The binding affinity of the separated oligomeric structures to type I collagen is dependent on the multimeric complex of the enzyme.

## 2. Materials and Methods

### 2.1. Generation of the MT1-MMP Constructs

All human MT1-MMP (UniProt accession number: P50281) constructs were cloned in the insect cell’s expression vector pAc5.1 (Thermo Fisher Scientific, Waltham, MA, USA). In the forward primers, the BiP signal sequence (MKLCILLAVVAFVGLSLG) was inserted to direct the recombinant protein into the cell culture media, and the reverse primers contained the His- and Strep-tags directly fused to the constructs to facilitate enzyme purification and detection. KpnI (at N-terminus) and NotI (at C-terminus) restriction enzymes were used to clone all constructs into the expression vector. WT-MT1-MMP (Ala^21^ to Ala^541^) and EA-MT1-MMP whole ectodomains were cloned using the primers MT1_F_BIP and pAC_MT1-MMP_His_Strep_R. Additionally, several more constructs were generated: L-Hpx1 (hemopexin domain with the linker) Gly^284^ to Cys^508^ was inserted with the primers pAc_Bip_Hpx_L_F and pAc_Hpx_Strep_R; Cat-L (catalytic domain with the linker) Ala^21^ to Gly^315^ was cloned using the primers MT1_F_BIP and pAC_Cat_L_His_Strep_R. Several sets of mutations were introduced into the furin cleavage site: R^108^RKR^111^ → AAAA (replacement of the entire furin cleavage site) with the primers Furin_D_MT1_F and Furin_D_MT1_R; R^111^ → A mutation was introduced with the primers MT1_R111A_F and MT1_R111A_R; R^89^ → A mutant was generated using the primers MT1_R89A_F and MT1_R89A_R. The sequences of primers can be found in the Supplementary Materials, Table S1. The mutations were introduced by site-directed mutagenesis standard protocols. The in-frame sequence of the constructs and the specific mutations were verified by DNA sequencing.

### 2.2. Expression, Purification, and Detection of MT1-MMP

The expression of the recombinant proteins in the insect cells was performed according to the manufacturer’s instructions (Thermo Fisher Scientific, Waltham, MA, USA). Briefly, Drosophila S2 cells were transiently or stably transfected, using the calcium phosphate method, with the constructs, and selected for 2 weeks with blasticidin. The stable transfected S2 cells were progressively scaled up to the desired volume in serum-free ex-cell medium. The conditioned medium was 10-fold concentrated and then applied to a 5 mL HiTrap IMAC (immobilized metal affinity chromatography) column. After washing the column with 50 mM sodium phosphate, 20 mM imidazole, 500 mM NaCl, pH 7.5, 10 µM CT1746 (was a gift from UCB/Celltech, Slough, UK) the elution of the His-tagged protein was performed using a linear gradient of imidazole (50 mM sodium phosphate, 250 mM Imidazole, 500 mM NaCl, pH 7.5, 10 µM CT1746). For Western blotting, the proteins were resolved on SDS-PAGE and then transferred to a PVDF membrane. After blocking the membrane with 5% milk for 1 h, the enzymes were detected with an anti-His-tag antibody conjugated with HRP.

### 2.3. Preparative Size-Exclusion Chromatography

Sephacryl S-200 gel filtration column (GE healthcare, Chicago, IL, USA) was used for all separation experiments with a bed volume of 120 mL connected to the AKTA FPLC purification system. The following running settings were used: flow rate: 0.5 mL/min; sample concentration: 1 mg/mL; total volume injected: 1 mL; fraction size: 1 mL, and 50 mM Tris pH 7.4, 150 mM NaCl, 5 mM CaCl_2_, 10 µM CT1746 as running buffer. Calibration of the column was performed by using Gel Filtration LMW and HMW Calibration Kit (GE Healthcare, IL, USA) with 50 mM Tris pH 7.4, and 150 mM NaCl as running buffer according to the manufacturer’s instructions. Briefly, four runs were performed as follows: blue dextran was used alone to determine the void volume of the column; in the second run a mixture of aldolase, carbonic anhydrase, and ribonuclease A was used; the third run had the composition of conalbumin, carbonic anhydrase, and ribonuclease A; in the mixture for the fourth run ovalbumin, carbonic anhydrase, and ribonuclease A were present. The IMAC-purified proteins were concentrated to approx. 1 mg/mL and passed through a 0.22 µm filter to remove any precipitated or aggregated enzyme before injecting it into the column. The enzymes concentrations were determined by using the Beer–Lambert Law. The absorption of the enzymes was measured at 280 nm, and the concentrations were calculated with their corresponding extinction coefficient [26]. The extinction coefficients calculated and used for each construct: 97,290 M^−1^cm^−1^ for WT-MT1-MMP-ED; 107,260 M^−1^cm^−1^ for R^111^ → A-WT-MT1-MMP-ED; 36,900 M^−1^cm^−1^ for WT-Cat-L; 46,870 M^−1^cm^−1^ for R^111^ → A-Cat-L; 61,880 M^−1^cm^−1^ for L-Hpx1.

### 2.4. Enzyme-Linked Immunosorbent Assay (ELISA)

Wells of a 96-well plate were coated with 10 µg/mL of type I collagen and incubated overnight at 4 °C. Blocking was done by adding 200 µL of 3% BSA followed by incubation with the WT-MT1-MMP-ED solution in a serial dilution for 1 h. The unbound enzyme was washed away and 50 µL of anti-His antibody conjugated with HRP was added to each well and allowed to bind for 1 h at 20 °C. A reporter enzyme’s substrate (tetramethylbenzidine) was added to each well and the chromogenic reaction was stopped by adding 2 N H_2_SO_4_. Endpoint measurement was recorded at 450 nm and the absorbance values were plotted against the antibody concentration. The measured points were fitted by non-linear least-squares regression [27]. All washing and dilution steps were performed with 50 mM Tris pH 7.4, 150 mM NaCl, 5 mM CaCl_2_, and 0.05% Tween (Sigma Aldrich, St. Louis, MO, USA).

### 2.5. Type I Collagen Proteolysis and Quenched Fluorescent Peptide Cleavage Assay

Assessment of the proper folding of the recombinant WT-MT1-MMP-ED was made using collagen cleavage assay in solution. The WT-MT1-MMP-ED expressed in the insect cells did not require activation, as the prodomain was cleaved off during the secretion in the conditioned media, unlike the refolded proMT1-MMP expressed in bacteria, which needs to be activated before cleavage assay. Type I collagen cleavage was performed in a final volume of 40 µL with 50 mM Tris pH 7.4, and 5 mM CaCl_2_ (Sigma Aldrich, St. Louis, MO, USA) as reaction buffer. 10 µg of type I collagen was incubated with various concentrations of WT-MT1-MMP-ED and incubated for 18 h at 25 °C. The cleavage reaction was loaded on an SDS gel followed by Coomassie brilliant blue staining.

The fluorometric assay was performed by using quenched fluorescent peptides QF-24 (Mca-Pro-Leu-Gly-Leu-Dpa-Ala-Arg-NH2) (Calbiochem, San Diego, CA, USA) as described earlier [28]. Briefly, QF-24 peptide substrate turnover rate was made in 100 µL final volume in 50 mM Tris pH 7.4, 150 mM NaCl, 10 mM CaCl_2_, 0.05% Brij 35. An amount of 10 µM QF-24 substrate was incubated with WT-MT1-MMP ectodomain and Cat-L construct, and the cleavage activity was monitored (λ_ex_ 330 nm, λ_em_ 390 nm) for up to 2 h using Tecan 200 plate reader. As specific inhibitors for MT1-MMP, 10 µM CT1746 or 250 mM TIMP-2 (expressed in insect cells) was added to the reaction.

## 3. Results

### 3.1. ProMT1-MMP Expressed in Drosophila S2 Cells Is Secreted in Its Active State

**Figure 1:**
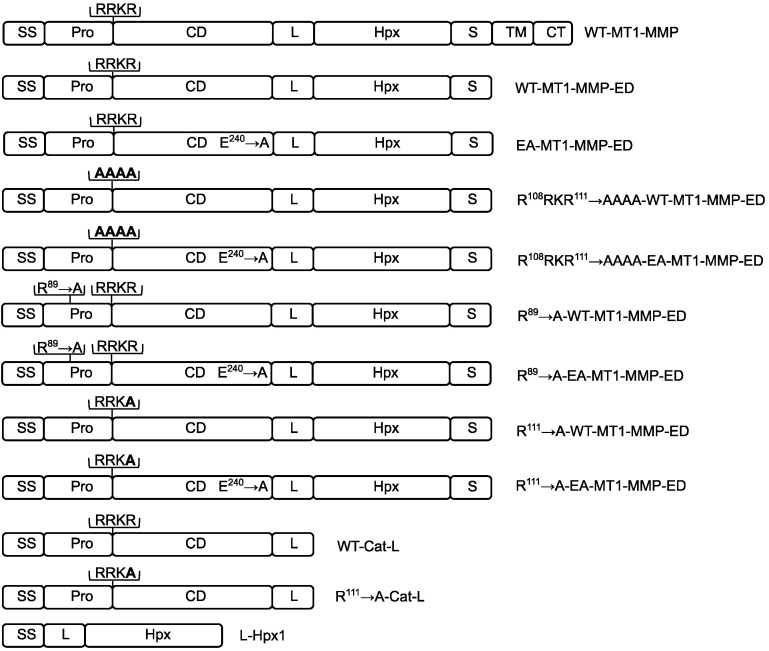
Schematic diagram of the WT-MT1-MMP domain structure and the truncated constructs used in the present study. The MT1-MMP domain configuration includes a signal sequence (SS), prodomain (Pro), catalytic domain (CD), linker (L), hemopexin-like domain (Hpx), stalk (S), transmembrane domain (TM), and the cytoplasmic tail (CT). RRKR represents the furin cleavage site. RRKA and AAAA are the mutated versions that block the removal of the prodomain. Position 89 is the second cleavage site in the mammalian cells and the R^89^ → A is the mutant used in this study.

**Figure 2:**
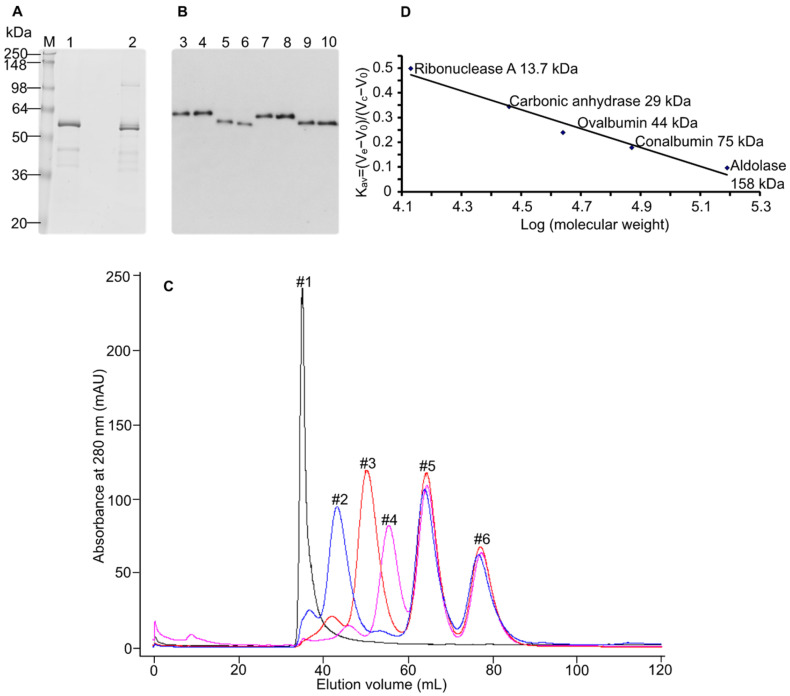
Mutations in the furin cleavage site prevent prodomain removal. (**A**) SDS-PAGE of the WT-MT1-MMP ectodomain (ED) expressed in drosophila S2 cells. WT-MT1-MMP ectodomain was stably transfected into drosophila S2 cells and the enzyme was purified on the IMAC column. The samples were loaded under reducing (with β-mercaptoethanol) (lane 1) and non-reducing conditions (without β-mercaptoethanol) (lane 2), M: molecular weight marker. (**B**) Western blot analysis of furin cleavage site MT1-MMP mutants. The mutants were transiently expressed in drosophila S2 cells and detected by Western blotting using an anti-His-tag antibody conjugated with HRP. Lane 3: R^108^RKR^111^ → AAAA-WT-MT1-MMP-ED; lane 4: R^108^RKR^111^ → AAAA-EA-MT1-MMP-ED; lane 5: R^89^ → A-WT-MT1-MMP-ED; lane 6: R^89^ → A-EA-MT1-MMP-ED; lane 7: R^111^ → A-WT-MT1-MMP-ED; lane 8: R^111^ → A-EA-MT1-MMP-ED; lane 9: WT-MT1-MMP-ED; lane 10: EA-MT1-MMP-ED. Two sets of mutants (R^111^ → A and R^108^RKR^111^ → AAAA) inhibited the cleavage of the prodomain from both WT-MT1-MMP-ED and EA-MT1-MMP-ED. (**C**) Chromatographic separation of the standard proteins used for the column calibration. The following markers have been used: peak #1 blue dextran—2,000,000 Da, peak #2 aldolase—158,000 Da, peak #3 conalbumin—75,000 Da, peak #4 ovalbumin—44,000 Da, peak #5 carbonic anhydrase—29,000 Da, peak #6 ribonuclease A—13,700 Da. The protein peaks were monitored at 280 nm absorption. (**D**) Calibration curve of sephacryl gel filtration column. The equation y = −0.3808x + 2.0455 derived from the calibration curve (representing the plotted K_av_ against log MW) was used to determine the molecular weight of the constructs. K_av_ (partition coefficient), V_e_ (elution volume), V_c_ (geometric column volume), and V_0_ (void volume).

Recombinantly produced MT1-MMP for oligomerization studies requires the enzyme to be properly folded. It is known that in the bacterial expression system, MT1-MMP can be expressed, but the refolding efficiency is at a very low level (about 10 % is folded, as measured by active site titration, data not shown). Therefore, we sought a eukaryotic expression system in which the MT1-MMP ectodomain can be produced and obtained in a properly folded structure. For this purpose, we employed the Drosophila Schneider 2 (S2) expression system by using the constitutive expression vector pAc 5.1. Initially, the whole ectodomain (Ala^21^ to Ala^541^) of WT-MT1-MMP and EA-MT1-MMP (the catalytic inactive mutant—E^240^ → A) were cloned with BiP signal sequence. To the C-terminus of each construct His- and Strep-tags were fused. (Figure 1). These constructs were stably transfected into S2 cells, expressed, and purified on an immobilized metal affinity chromatography (IMAC) column. Although the WT-MT1-MMP ectodomain was transfected with the cDNA containing the prodomain, SDS-PAGE analysis of the purified WT-MT1-MMP-ED showed that the size of the enzyme is slightly smaller than expected (Figure 2A). This result suggested that the prodomain of the WT-MT1-MMP-ED might be removed during secretion from the insect cells into the cell culture medium. To test this hypothesis, several mutations were introduced by site-directed mutagenesis in the furin cleavage site of the WT-MT1-MMP and EA-MT1-MMP ectodomains: R^108^RKR^111^ → AAAA (replacement of the whole furin cleavage sequence) and R^89^ → A (the second cleavage site in the mammalian cells [8]). The constructs were transiently expressed in S2 cells, and the recombinant enzymes were detected in the conditioned medium with an anti-His-tag antibody. Western blot analysis showed that mutagenesis of the furin cleavage sequence (R^108^RKR^111^ → AAAA) led to the retention of the prodomain both in the WT-MT1-MMP-ED and EA-MT1-MMP-ED (Figure 2B, lanes 3 and 4) when compared with the unmutated furin sequence of the same constructs (Figure 2B, lanes 9 and 10). R^89^ → A mutation, in the second cleavage site, did not prevent prodomain removal (Figure 2B, lanes 5 and 6). To minimize any structural disturbance of proMT1-MMP caused by mutating the entire furin cleavage site, only one mutation was introduced at position 111 (R^111^ → A). Expression of this mutant in S2 cells revealed that this particular substitution was sufficient to abrogate the cleavage of the prodomain from the enzyme (Figure 2B, lanes 7 and 8); therefore, this construct was chosen for further analysis of the proMT1-MMP. Moreover, to prove that the prodomain is cleaved off at the furin cleavage site, the purified soluble enzyme was N-terminally sequenced (data not shown), and the results indicated that, indeed, the processed soluble WT-MT1-MMP-ED started with the Y^112^AIQGL amino acids sequence corresponding to the sequence immediately after the furin site (R^109^RKR^111^). Hence, the whole ectodomain of the WT-MT1-MMP construct, expressed in insect cells, is present in the active structural form in the solution.

### 3.2. The Prodomain of proMT1-MMP Inhibits the Oligomerization

**Figure 3:**
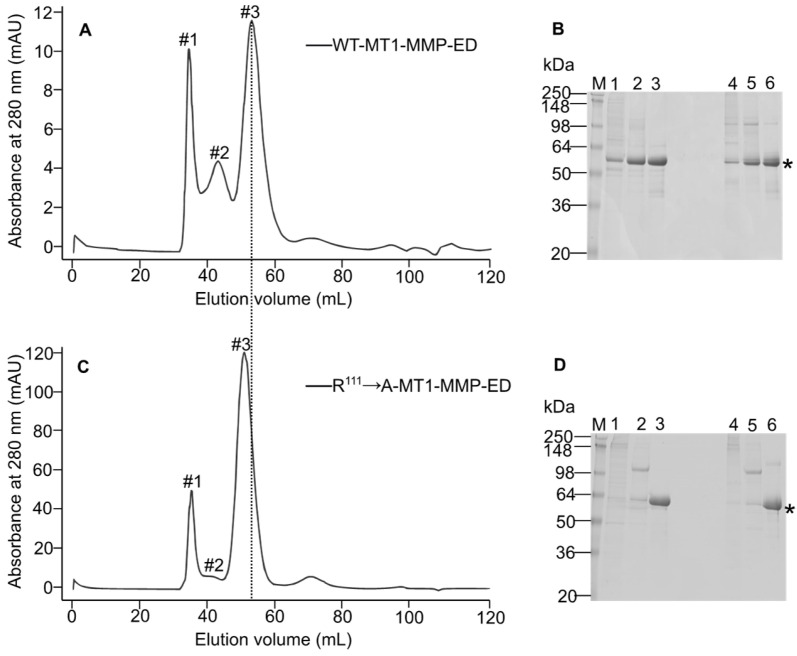
MT1-MMP furin cleavage site mutant (R^111^ → A) is monomeric, while the WT is multimeric. Analysis of the oligomeric structures of WT and mutant MT1-MMP by size-exclusion chromatography and SDS-PAGE. (**A**) Gel filtration elution profiles of WT-MT1-MMP ectodomain. After purification by affinity chromatography, the enzyme solution was concentrated at 1 mg/mL and applied to a sephacryl 200 gel filtration column. On the chromatogram is marked with #1 for the first peak (the highest molecular weight—oligomer), #2 for the second peak—dimer, and #3 for the third peak—monomer. (**B**) Coomassie brilliant blue-stained SDS-PAGE characterization of the peaks content from gel filtration column of the WT-MT1-MMP-ED. M-molecular weight marker; lane 1: WT-MT1-MMP-ED #1; lane 2: WT-MT1-MMP-ED #2; lane 3: WT-MT1-MMP-ED #3; lanes 1 to 3 under reducing conditions; lanes 4 to 6 in the same order as for lanes 1 to 3, but under non-reducing conditions. (**C**) Gel filtration elution profile of R^111^ → A-MT1-MMP ectodomain. The same procedure and labeling as in (**A**). (**D**) Coomassie brilliant blue-stained SDS-PAGE analysis of R^111^ → A-MT1-MMP-ED from gel filtration. M: molecular weight marker; lane 1: R^111^ → A-MT1-MMP-ED #1; lane 2: R^111^ → A-MT1-MMP-ED #2; lane 3: R^111^ → A-MT1-MMP-ED #3; lanes 1 to 3 under reducing conditions; lanes 4 to 6 in the same order as for lanes 1 to 3, but under non-reducing conditions. The asterisks indicate the recombinant MT1-MMP-ED. The dotted line points out the shift of R^111^ → A-MT1-MMP-ED mutant to a slightly higher molecular weight due to the presence of the prodomain.

To investigate the influence of the prodomain presence on the structure of the enzyme, WT-MT1-MMP-ED and R^111^ → A-MT1-MMP-ED were stably transfected into S2 cells and scaled up so that the enzymes were present in sufficient quantity for study. The constructs generated were purified from the insect cell culture supernatant by IMAC and then applied to size-exclusion chromatography. Before running the samples on the sephacryl column, the concentrated enzymes were passed through a 0.22 µm filter to remove any precipitated or aggregated proteins which may have occurred during purification and concentration, thus avoiding the unwanted protein multimerization provided by the aggregated enzymes. Elution of WT-MT1-MMP ectodomain preparations on the sephacryl column yielded three distinct peaks (marked with #1, #2, and #3; Figure 3A). Coomassie brilliant blue-stained SDS gel analysis revealed the presence of WT-MT1-MMP-ED in all fractions. Using the calibration curve of the sephacryl S200 column (Figure 2C,D), it can be estimated that peak #3 has a size of 60 kDa that corresponds to the monomeric form of WT-MT1-MMP-ED. Peak #2 also contains WT-MT1-MMP-ED, and its size is calculated to be 120 kDa. The first peak (#1) contains the highest molecular weight forms of WT-MT1-MMP-ED, but due to the resolution of the gel filtration column, it is not possible to calculate the exact multimeric state of the enzyme beyond the dimeric form. The samples corresponding to each peak were loaded on SDS-PAGE (Figure 3B). Under reducing and non-reducing conditions, the WT-MT1-MMP-ED migrated at the same level, suggesting that the multimeric structures are built up based on non-covalent interactions. On the other hand, the R^111^ → A-WT-MT1-MMP-ED (the furin mutant version) chromatogram showed only two major peaks (Figure 3C). SDS-PAGE analysis of all fractions from the gel filtration column revealed that this mutant is present in the third peak only (Figure 3D, lane 3). Peak #1(Figure 3D, lane 1) and peak #2 (Figure 3D, lane 2) contained protein contaminants originating from the insect cell culture medium. Determination of the molecular weight of the enzyme from peak #3 provided a size of 70 kDa. The difference of 10 kDa in the molecular weight between this furin mutant and the WT enzyme indicates the presence of the prodomain, and the enzyme adopts a monomeric structure. This molecular mass difference can be observed as a shift of the third peak towards a higher molecular weight (Figure 3, marked with a dotted line).

### 3.3. The Catalytic Domain Is Involved in the Homomultimerization of MT1-MMP

**Figure 4:**
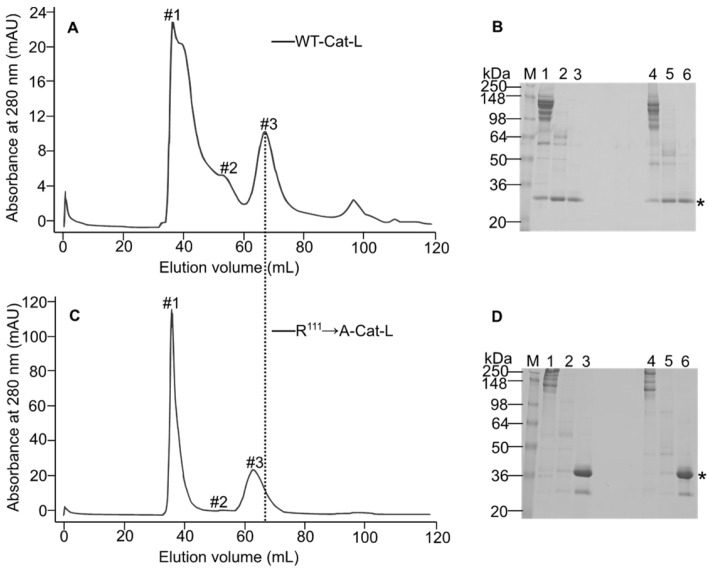
MT1-MMP catalytic domain is involved in the formation of the oligomeric structures. (**A**) Gel filtration elution chromatogram of WT-Cat-L truncated version. The recombinant enzyme was purified by affinity chromatography (IMAC) and then loaded on a sephacryl column. Peak #1 is the highest molecular oligomer, peak #2 is dimeric structure, and peak #3 is monomeric form. (**B**) Coomassie brilliant blue-stained SDS-PAGE profile of the eluted WT-Cat-L oligomers. M: molecular weight marker; lane 1: WT-Cat-L #1; lane 2: WT-Cat-L #2; lane 3: WT-Cat-L #3; lanes 1 to 3 under reducing conditions; lanes 4 to 6 in the same order as for lanes 1 to 3, but under non-reducing conditions. (**C**) Size-exclusion chromatography profile of R^111^ → A-Cat-L. The same procedure and labeling as in (**A**). (**D**) Coomassie brilliant blue-stained SDS-PAGE of the separated R^111^ → A-Cat-L. M: molecular weight marker; lane 1: R^111^ → A-Cat-L #1; lane 2: R^111^ → A-Cat-L #2; lane 3: R^111^ → A-Cat-L #3; lanes 1 to 3 under reducing conditions; lanes 4 to 6 in the same order as for lanes 1 to 3, but under non-reducing conditions. The asterisks indicate the recombinant truncated versions of MT1-MMP. The dotted line points out the shift of R^111^ → A-Cat-L mutant to a slightly higher molecular weight due to the presence of the prodomain.

**Figure 5:**
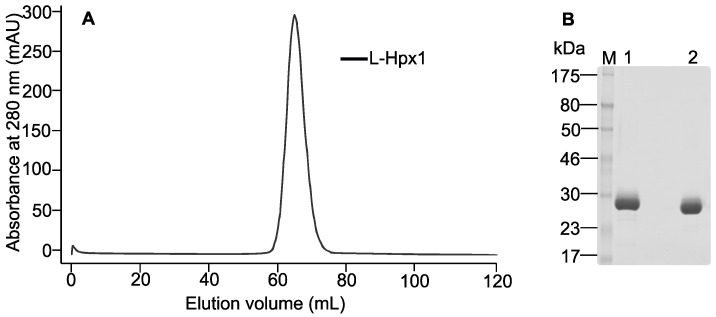
The hemopexin domain of MT1-MMP is monomeric. (**A**) Size-exclusion chromatography elution profile of recombinant L-Hpx1 domain. (**B**) Coomassie brilliant blue-stained SDS-PAGE analysis of L-Hpx1 eluted fraction from gel filtration. M: molecular weight marker; lane 1: disulfide bond reduced L-Hpx1 domain; lane 2: non-reduced L-Hpx1 domain.

To further dissect the domain contribution to the oligomerization process of MT1-MMP, several constructs were generated: WT-Cat-L (catalytic domain with the linker), R^111^ → A-Cat-L (furin cleavage site mutant with the catalytic domain and the linker), and L-Hpx1 (hemopexin domain with the linker). These constructs were expressed in the insect cells and purified by IMAC. Size-exclusion chromatography of the WT-Cat-L construct showed several peaks, marked with #1, #2, and #3 on the chromatogram (Figure 4A). All fractions were loaded on SDS-PAGE, and staining with Coomassie brilliant blue revealed that all three peaks contained the truncated WT enzyme (Figure 4B). The calculated molecular weight of peak #3 provided a size of 24 kDa, which corresponds to the monomeric form of the catalytic domain with the linker, but without the prodomain. Peak #2 has a molecular weight of approx. 49 kDa, which is the size of the calculated dimeric form of this construct. The eluted fractions between peak #1 and peak #2 contain the recombinant Cat-L (Figure 4B), but there was no distinctive peak for a trimer structure. In this region of the chromatogram, molecules that are also larger than a trimer are eluted, which suggests that the Cat-L construct might form higher multimeric structures than a trimer. In the case of the R^111^ → A-Cat-L construct, only two peaks can be seen on the gel filtration elution profile (Figure 4C). Loading all fractions between peak #1 and peak #3 on SDS-PAGE gel revealed that this truncated construct is present in the third peak only and is absent in the others, suggesting again that the presence of the prodomain interferes with the oligomerization. The molecular weight of this construct, derived from the calibration curve, is 34 kDa. Examination of the L-Hpx1 domain on gel filtration (Figure 5A) showed one peak with a size of 26.3 kDa, indicating that this construct, expressed in the insect cells, has a monomeric structure. SDS-PAGE analysis confirmed that L-Hpx1 is properly folded, as it can be observed under non-reducing conditions when the protein runs at a lower molecular weight compared with the reduced form (Figure 5B). This is due to the correct formation of the disulfide bond in the hemopexin domain, providing the protein a more compact structure.

### 3.4. MT1-MMP Oligomeric Structures Bind with Different Affinities to Type I Collagen

**Figure 6:**
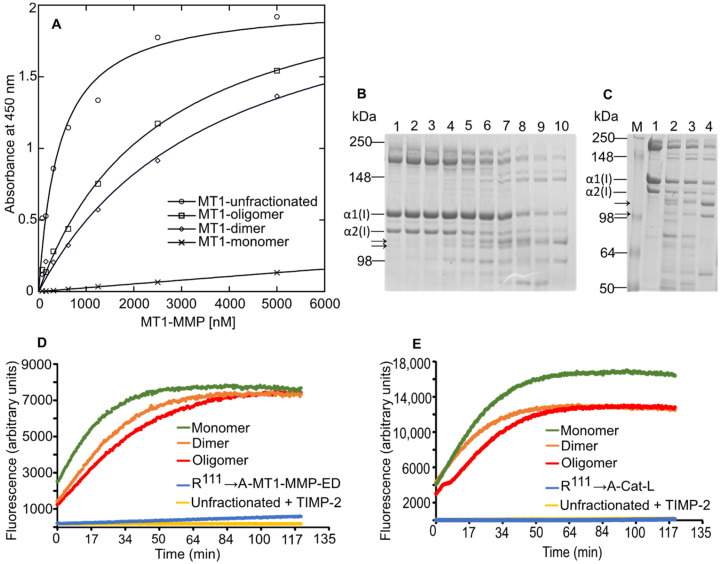
The multimeric structures of MT1-MMP bind to type I collagen with different affinities. (**A**) Measurement of WT-MT1-MMP-ED binding to type I collagen in ELISA. The wells of an ELISA plate were coated with 10 μg/mL type I collagen from rat tail. Serial dilutions of WT-MT1-MMP-ED were applied to the coated wells and incubated at 20 °C. The binding of WT-MT1-MMP-ED to collagen was detected with an anti-His-tag antibody conjugated with HRP. (**B**) The collagenolytic activity of unfractionated WT-MT1-MMP ectodomain. 10 μg of rat tail type I collagen was incubated with various concentrations of WT-MT1-MMP-ED and the cleavage reaction was analyzed after 18 h post-incubation in Coomassie brilliant blue-stained SDS-PAGE. Lane 1: type I collagen without enzyme; lane 2: WT-MT1-MMP-ED incubated with CT1746 inhibitor; lanes 3 to 10: increasing concentration of WT-MT1-MMP-ED (0.5, 1, 2, 4, 8, 16, 32 and 64 nM). (**C**) The collagenolytic activity of the separated multimeric forms of WT-MT1-MMP-ED. 10 μg of rat tail type I collagen was incubated with 10 nM of the WT-MT1-MMP-ED oligomeric forms for 18 h and then loaded on SDS-PAGE. M: molecular weight marker; lane 1: type I collagen alone; lane 2: oligomeric WT-MT1-MMP-ED; lane 3: dimeric WT-MT1-MMP-ED; lane 4: monomeric WT-MT1-MMP-ED. The upper arrow represents a ¾ fragment of the α1 chain and the lower arrow represents the ¾ fragment of the α2 chain collagenolytic products. (**D**) QF-24 peptide substrate hydrolysis by WT-MT1-MMP-ED oligomeric forms. 20 nM of multimeric structures were incubated with 10 µM QF-24 fluorogenic substrate and the hydrolysis was monitored for 2 h at 27 °C. 250 nM TIMP-2 has been used as a specific endogenous inhibitor of MT1-MMP. (**E**) QF-24 peptide substrate cleavage by Cat-L multimeric complexes. The same procedure was used as in (**D**).

To investigate whether there are any functional consequences of WT-MT1-MMP-ED multimerization, the binding of the oligomeric forms of WT-MT1-MMP-ED to type I collagen was analyzed in ELISA, and the degradation of this substrate was performed. Measurements of the binding in ELISA of the monomeric form of WT-MT1-MMP-ED showed that this structure has a weak affinity towards type I collagen compared to the unfractionated WT-MT1-MMP-ED (all oligomeric forms of WT-MT1-MMP-ED in the same sample), which provided the highest signal in ELISA (Figure 6A). The dimeric and higher oligomeric forms of WT-MT1-MMP-ED showed a binding affinity higher than the monomeric form but not as high as the unfractionated. Next, the enzymatic activity of all oligomeric structures was measured by their ability to degrade type I collagen in solution. Thus, the unfractionated WT-MT1-MMP-ED was able to degrade type I collagen to ¾ specific fragments in a concentration dependence manner and CT1746, a synthetic matrix metalloproteinase inhibitor, could block the degradation activity of the unfractionated WT-MT1-MMP-ED (Figure 6B). Incubation of the separated multimeric structures with type I collagen, followed by migration on SDS-PAGE, revealed that all multimeric forms of WT-MT1-MMP-ED were able to cleave type I collagen substrate with high efficiency (Figure 6C). Furthermore, we have analyzed the ability of the oligomeric forms of WT-MT1-MMP-ED to hydrolyze the fluorogenic peptide substrate QF-24. All multimeric structures of WT-MT1-MMP ectodomain (Figure 6D) and Cat-L construct (Figure 6E) showed cleavage of the fluorogenic peptide. The specificity of the substrate cleavage was investigated by using the TIMP-2 endogenous inhibitor of MT1-MMP. In this regard, the experimental results indicate that the hydrolysis of the substrate was inhibited in all MT1-MMP multimeric structures (Figure 6D,E) in the presence of TIMP-2. Moreover, the CT1746 synthetic inhibitor completely blocked the cleavage activity of the multimeric structures. These data demonstrate the presence and the identity of the MT1-MMP in the eluted fractions.

## 4. Discussion

The biological functions of MT1-MMP are regulated and controlled by various cellular and molecular pathways. Among these regulatory mechanisms, oligomerization of the MT1-MMP controls some of its biological functions. Thus, dimerization through the hemopexin domain is involved in the activation of the proMMP-2. In this process, it is postulated that one monomer of MT1-MMP interacts with the TIMP-2 molecule, and the proMMP-2 molecule binds to the C-terminal domain of TIMP-2, forming a ternary complex, and then the second MT1-MMP monomer cleaves the proMMP-2 to generate its active form [10,21]. The second mechanism of dimerization involves the transmembrane domain of MT1-MMP, and is important for proMMP-2 activation [20]. Although both dimerization domains are involved in proMMP-2 activation and collagen proteolysis, transmembrane-dependent dimerization is mainly responsible for proMMP-2 activation, while hemopexin-mediated dimerization is required for collagenolysis [20]. Hence, oligomerization has interesting functional consequences, and in the present study we aimed to further investigate the mechanism of MT1-MMP oligomerization by using recombinant MT1-MMP heterologously expressed in eukaryotic cells, so that the ectodomain and its truncated versions are properly folded and of high quality. In this context, we showed that the whole ectodomain of MT1-MMP can form multimeric structures. Initial experimental data of MT1-MMP ectodomains, recombinantly expressed in insect cells, showed that the enzyme had a slightly smaller size on SDS-PAGE, as expected for the whole ectodomains, when compared with the same construct expressed in bacterial cells. We hypothesized that during the secretion of the enzyme from the insect cells the prodomain is removed, unlike the MT1-MMP expressed in bacteria, where the prodomain is attached and needs to be activated. In this regard, several reports have demonstrated that MT1-MMP is activated by furin-like protein convertases at two potential furin sequence motifs: R^89^RPR^92^↓C^93^ and R^108^RKR^111^↓Y^112^ [8,29]. To test our hypothesis, we have introduced several mutations in the two furin sequence motifs. Mutations introduced in the primary furin cleavage site (R^108^RKR^111^) prevented the removal of the prodomain. In contrast, the mutation in the second furin sequence motif did not block the cleavage of the prodomain, suggesting that the insect cells cannot activate MT1-MMP at this site. Expression of MT1-MMP in another type of non-mammalian eukaryotic cell, such as yeast, showed that the secreted MT1-MMP can be found both as the activated and zymogen forms, and the activation is an autocatalytic event [30]. In our insect cell expression system, the MT1-MMP is secreted in the active form, and based on WT-MT1-MMP-ED and EA-MT1-MMP-ED, our results indicate that the removal of the prodomain is not an autocatalytic process, but rather it happens during secretion, and it is likely processed by proprotein convertase(s) of the insect cell. Otherwise, the prodomain of EA-MT1-MMP-ED (catalytically inactive mutant) would have not been removed. On the other hand, the furin cleavage site mutant (R^111^ → A-WT-MT1-MMP-ED) had a different elution profile compared with the WT-MT1-MMP-ED version on size-exclusion chromatography. Interestingly, the absence of the furin mutant enzyme in the dimeric and oligomeric structures suggests that the presence of the prodomain on the enzyme interferes with the oligomerization of the MT1-MMP. Another report describes the expression of MT1-MMP-ED in insect cells with the baculovirus system [31]. Unlike that work, we were able to purify the MT1-MMP-ED by using IMAC. One of the explanations could be that we used the synthetic inhibitor CT1746 during the entire process of purification and separation, thus minimizing the autodegradation of the enzyme on the column.

We were also interested in further dissecting the multimerization mechanism of MT1-MMP. Thus, the catalytic domain with the linker of MT1-MMP was able to multimerize. Interestingly, this truncated version of MT1-MMP is assembled in higher molecular complexes than the trimeric structure. In line with this observation, another report proposed that MT1-MMP could be found on the cell surface as a tetrameric complex [21]. The furin cleavage site mutant of the same truncated construct was found only in a monomeric state, suggesting, again, that the prodomain blocks the multimeric assembly of MT1-MMP. These results, in addition, indicate that the catalytic domain is sufficient for MT1-MMP ectodomain oligomerization. Reports showed that MT1-MMP can form dimers via the hemopexin domain [10], transmembrane domain [20], or cytoplasmic tail [22]. In this context, our hemopexin domain with the linker construct, expressed in insect cells, did not produce a homodimeric structure. There are conflicting reports which show that the hemopexin domain does not form homodimers [32,33], while another one indicates that MT1-MMP forms dimers through the hemopexin domain [10]. These opposing differences might come from the experimental setups. In our case, we can rule out the potential misfolding of the recombinant protein, since the hemopexin domain was expressed in eukaryotic cells which produced a properly formed disulfide bond as demonstrated with the oxidized and reduced state of the recombinant protein. As we expressed only the extracellular domain of MT1-MMP, we did not investigate the other domains (transmembrane and intracellular) mediated oligomerization. However, from our experimental results, it appears that only the catalytic domain of the enzyme is necessary and sufficient to multimerize the extracellular domain of the MT1-MMP, since the linker does not seem to contribute to the oligomerization, as both the catalytic domain and the hemopexin domain had the linker, and the oligomeric complexes are built up only through the catalytic domain.

As MT1-MMP is considered to be a major collagenolytic enzyme in vivo, we were interested in analyzing the functional consequences of the oligomerization on collagen. Interestingly, the binding affinity of MT1-MMP to type I collagen was dependent on its oligomeric state. Thus, the highest binding signal in ELISA was provided by the unfractionated sample. Unexpectedly, the monomeric structure of MT1-MMP has a weak binding affinity towards type I collagen, while the affinity increased with the increasing complexity of the multimeric structure of MT1-MMP. One simple explanation would be that in the higher order of oligomerization there is an avidity effect on binding compared with the monomeric form. These results suggest that efficient and strong binding of MT1-MMP to type I collagen requires multimeric assembly. On the other hand, the collagenolytic activity showed that the MT1-MMP ectodomain can cleave the collagen in solution in a concentration-dependence manner. Although the monomeric form of MT1-MMP has weak binding to type I collagen, it is highly active in collagen degradation and it is comparable with the other oligomeric structures of MT1-MMP. Furthermore, we were interested in proving the MT1-MMP identity in the fractions corresponding to the multimeric forms. The results revealed that all oligomeric forms of WT-MT1-MMP-ED and Cat-L hydrolyzed the fluorogenic substrate. To further demonstrate that the cleaved substrate is due to the presence of MT1-MMP in the sample, we have used the TIMP-2 endogenous inhibitor of MT1-MMP. Thus, the cleavage of the substrate, in all multimeric structures, was inhibited by TIMP-2 (Figure 6D,E), proving, once again, the presence of MT1-MMP in all oligomeric forms and the specificity of the proteolytic activity.

In conclusion, we have identified new structural features and functions of MT1-MMP. Structurally, the oligomeric assembly of MT1-MMP ectodomain, recombinantly expressed in eukaryotic cells, is only dependent on the catalytic domain, and the unremoved prodomain interferes with the multimerization process. Functionally, the binding affinity to type I collagen is dependent on the multimeric state of MT1-MMP and all oligomeric forms of MT1-MMP ectodomain cleave type I collagen with comparable efficiency.

## Data Availability

Not applicable.

## Supplementary Materials

The following supporting information can be downloaded at: https://www.mdpi.com/article/10.3390/biom12081145/s1, Table S1: Primers used in the cloning process.
