# Supplementary material for: The Catalytic Domain Mediates Homomultimerization of MT1-MMP and the Prodomain Interferes with MT1-MMP Oligomeric Complex Assembly
Source: Biomolecules. 2022 Aug 19;12(8):1145. doi: 10.3390/biom12081145 (PMC9406036; doi:10.3390/biom12081145)
Supplement: Supplementary file 1 [file biomolecules-12-01145-s001.zip › biomolecules-1835392-supplementary.pdf]

Table S1. Primers used in the cloning process.

Primer name	Primer sequence
MT1_F_BIP	5′-GTTAGCGCATAGGGTACCATGAAGTTATGCATATTACTGGCCGT CGTGGCCTTTGTTGGCCTCTCGCTCGGGGCGCTCGCCTCCCTCGGCTCG-3′
pAC_MT1-MMP_His_Strep_R	5′-GTAGCGGATATGGCGGCCGCTCATTATTTTTCGAACTGCGGGT GGCTCCAATGGTGATGGTGATGGTGGGCAGCCGCGCTCACCGCCCC-3′
pAc_Bip_Hpx_L_F	5′-CACCCGAGCCGAGGTACCATGAAGTTATGCATATTACTGGCCGT CGTGGCCTTTGTTGGCCTCTCGCTCGGAGGGGGTGAGTCAGGGTTCCCCACC-3′
pAc_Hpx_Strep_R	5′-GTAGCGGATATGGCGGCCGCTCATTATTTTTCGAACTGCGGGT GGCTCCAGCAGCCCATCCAGTCCCTCAG-3′
MT1_R111A_F	5′-GCTGAGATCAAGGCCAATGTTCGAAGGAAGGCTTACGCCATCC AGGGTCTCAAATGG-3′
MT1_R111A_R	5′-CCATTTGAGACCCTGGATGGCGTAAGCCTTCCTTCGAACATTGG CCTTGATCTCAGC-3′
MT1_R89A_F	5′-GATGCAGACACCATGAAGGCCATGGCCCGCCCCCGATGTGGTGTTCCAG AC-3′
MT1_R89A_R	5′-GTCTGGAACACCACATCGGGGGCGGGCCATGGCCTTCATGGTGTCTGCA TC-3′
pAC_Cat_L_His_Strep_R	5′-AACCTGGTCTTGGCGGCCGCCTATTATTTTTCGAACTGCGGGTGGCTCCA GTGATGGTGATGGTGATGCCCATAGGTGGGGTTTTTGGG-3′
Furin_D_MT1_F	5′-GCTGAGATCAAGGCCAATGTTGCTGCCGCAGCGTACGCCATCCAGGGTCT CAAATGG-3′
Furin_D_MT1_R	5′-CCATTTGAGACCCTGGATGGCGTACGCTGCGGCAGCAACATTGGCCTTGA TCTCAGC-3′
